# Characterizing the composition, metabolism and physiological functions of the fatty liver in *Rana omeimontis* tadpoles

**DOI:** 10.1186/s12983-019-0341-x

**Published:** 2019-11-14

**Authors:** Wei Zhu, Meihua Zhang, Liming Chang, Wenbo Zhu, Cheng Li, Feng Xie, Huan Zhang, Tian Zhao, Jianping Jiang

**Affiliations:** 1 CAS Key Laboratory of Mountain Ecological Restoration and Bioresource Utilization & Ecological Restoration Biodiversity Conservation Key Laboratory of Sichuan Province, Chengdu Institute of Biology, Chengdu, 610041 China; 2 University of Chinese Academy of Sciences, Beijing, 100049 China; 3 State Key Laboratory of Integrated Management of Pest Insects and Rodents, Institute of Zoology, Chinese Academy of Sciences, Beijing, 100101 China

**Keywords:** Hepatic fat deposition, Fatty liver, Metabolic profiling, Metabolism, Metamorphosis

## Abstract

**Background:**

Fat storage is required for the life cycle of many organisms. The primary fat depot for most vertebrates is white adipose tissue. However, in primitive vertebrates (e.g., agnathan group and elasmobranchs), the liver is usually responsible for fat storage. Among the vertebrates, amphibians have a unique status, as their larvae live in the water and exhibit some primitive traits that are similar to fish. Although it has been recognized that adult frogs use their abdominal white adipose tissue as a primary fat depot, how tadpoles store their fat is still inconclusive. The metabolic traits and physiological functions of primitive fat depots may have wide-ranging implications on the pathology of abnormal lipid deposition in mammals and the evolution of fat storage.

**Results:**

*Rana omeimontis* tadpoles used their liver as the primary fat depot. In sufficiently fed tadpoles at stage 30–31, the hepatosomatic index (HSI) reached up to 7%, and triglycerides (TG) accounted for 15% of liver weight. Their liver resembled white adipose tissue in histological morphology, characterized by polygonal hepatocytes filled with fat. Their liver metabolic composition was unique, characterized by the dominance of maltotriose, arachidonic acid and dipeptides in soluble carbohydrates, free fatty acids and amino acids. Hepatic fat was the major metabolic fuel of fasted *R. omeimontis* tadpoles, which had similar reserve mobilization and allocation patterns as mammals. From a developmental perspective, hepatic fat was important to fuel late metamorphic climax. Interestingly, starvation induced accelerated metamorphosis in tadpoles with high HSI (4.96 ± 0.21%). However, this phenomenon was not observed in tadpoles with low HSI (2.71 ± 0.16%), even though they had similar initial body weight and developmental stage. Hepatic fat abundance was the most prominent difference between the two groups.

**Conclusion:**

To the best of our knowledge, this is the first report that liver can be the primary fat depot in vertebrates with higher evolutionary status than bony fish. The unique hepatic histological and metabolic traits likely either guard their liver against lipotoxicity or make their hepatocytes adapt to fat accumulation. This fatty liver could be a primitive counterpart of mammalian white adipose tissue (WAT). In addition, our study showed that the hepatic reserves of tadpoles, especially TG content, may provide body condition signals to modulate metamorphosis.

## Background

Fat storage is required for the life cycle of many organisms. Primitive vertebrates (e.g., the agnathan group), elasmobranchs (e.g., sharks) and some bony fish (e.g., cod, coley and flatfish) synthesize and store most of their body fat in their liver [1–6]. In other bony fish, amphibians, reptiles, birds and mammals, even though liver is still responsible for lipogenesis, white adipose tissue (WAT) appears and replaces liver to become their primary fat depot [2, 7–10]. Liver can still be a secondary fat depot in fish, amphibians and reptiles [8, 9, 11–13], but hepatic lipid deposition in mammals and humans may be associated with increased risk of liver injuries [14, 15]. Accordingly, fat deposition in the liver is likely a primitive trait of vertebrate evolution [2]. From this point of view, the metabolic traits and physiological functions of the primitive fatty liver may have wide-ranging implications on the pathology of abnormal lipid deposition in humans and shed light on the driving force behind evolution of fat depot [15].

Although it has been recognized that the fat body, the major abdominal WAT of amphibians, is the primary fat depot in adult frogs [8, 9, 16, 17], little attention has been paid to the fat storage of tadpoles. Some previous studies indicated that the fat body could also be the major fat depot in tadpoles (e.g., *Lithobates catesbeianus* and *R. curtipes*) [9, 18–22]. They observed that it can provide energy to tadpoles during starvation, and it is also necessary for sustaining energy production at the late metamorphic climax of tadpoles when apoptotic tissues have been absorbed [23]. A recent study even suggested that the fat body might play critical roles in determining whether metabolic reserves meet the nutrient requirements of metamorphic climax of tadpoles [24]. The fat level of metamorphosizing tadpoles is associated with the survival strategies of froglets and the survival rate and reproductive capacity of adults [25, 26]. Despite the significance of fat body during tadpole metamorphosis, some frog species, e.g., *Microhyla fissipes*, *R. chaochiaoensis*, *R. omeimontis* and *Bufo melanostictus*, are devoid of fat-accumulating fat bodies across their larval stages (our observation and [18]). Surprisingly, in our preliminary studies, we found that *R. omeimontis* tadpoles store fat mainly in their livers, whose histological structure resembles that of adipose tissue if food is available. Although hepatic fat accumulation has also been observed in overwintering frogs [27–29], their liver is the major organ for triglyceride synthesis but a secondary site for triglyceride deposition [9]. To the best of our knowledge, the fatty liver of *R. omeimontis* tadpoles is the first observation that liver can be a primary fat depot in amphibians, or indeed in any animals with higher evolutionary status than bony fish. Importantly, these tadpoles likely experience a transition of the fat depot from liver to the fat body after metamorphosis. Such a phenomenon makes them unique models for investigating the regulation mechanisms of fat deposition.

Currently, we know little about the metabolism and physiology of amphibians’ use of liver as the primary fat depot. The most basic knowledge is the metabolic composition of their fatty liver. In humans and other mammals, hepatic lipid deposition is associated with increased risk of hepatic injury and steatohepatitis due to lipotoxicity [30–32]. It has been recognized that the lipid types that accumulate in liver, rather than their total amount, are tightly associated with the occurrence of lipotoxicity [33, 34]. For example, the accumulation of saturated free fatty acid (FFA) and diacylglycerol likely increases the probability of lipotoxicity, while a high ratio of monounsaturated FFAs to saturated FFAs is a sign of a low probability of lipotoxicity [33, 34]. Since hepatic fat deposition is a natural biological process in *R. omeimontis* tadpoles, these tadpoles are likely avoiding liver lipotoxicity. Uncovering the metabolic profile of their liver may be valuable in verifying and extending our knowledge of liver injuries caused by hepatic steatosis. Another basic question is whether and how metabolism differs between *R. omeimontis* tadpoles and animals with extrahepatic fat depots, as well as between *R. omeimontis* tadpoles and other animals with only a hepatic fat depot. Since liver is the common depot of glycogen, amino acids and lipids of *R. omeimontis* tadpoles, it would be interesting to know how their hepatic reserves are metabolized during starvation. The order of reserve mobilization during starvation is an important metabolic trait of animals. It varies between species, with potential implications for the physiological significance of metabolic reserves and their storage organs [35]. In elasmobranchs, amino acids and carbohydrates are preferentially mobilized over lipids, partly due to the role of hepatic fat in providing hydrostatic lift [36]. However, hepatic fat is preferentially consumed over glycogen and tail proteins in Atlantic cod (*Gadus morhua*) [37]. The differential usage of metabolic fuels between organs is also an important aspect of catabolism in animals. For example, glucose and ketone bodies synthesized in the liver are consumed by numerous organs except the liver in fasted mammals, while FFAs released from WAT are mainly used in liver and muscle for energy production. These metabolic traits in naturally fatty livers may be meaningful in understanding the metabolic disorder in human fatty liver disease. It is also important to know whether hepatic fat has physiological functions similar to those of the fat body, especially with respect to fueling and regulating metamorphosis. Answering these questions may deepen our understanding of amphibian physiology and provide insight into the functional evolution of fat depot.

To answer the questions mentioned above, we first observed fat deposition in the *R. omeimontis* tadpoles. Then, we investigated the metabolic profiles and the metabolic pattern of hepatic reserves during starvation by metabolomics. Last, we investigated the role of hepatic lipids during tadpole metamorphosis.

## Materials and methods

### Animal sampling and culture

*R. omeimontis* is distributed in plain, hill and mountain areas (altitude 250–2100 m. a. s. l.) in Sichuan, Gansu, Chongqing, Hunan, Guizhou and Hubei provinces in China [38]. Their breeding seasons are from the end of August to October ([38] and our observation). Their tadpoles live across winter and metamorphosize in the next May to July [38]. In this study, ten egg clutches of *R. omeimontis* were collected in October at Anzihe Natural Reserve (103.459885° E, 30.744614° N, 701 m) in Sichuan Province, China. These clutches (ranging from 400 to more than 1000 eggs) were placed in aquatic containers (42 × 30 × 10 cm, water depth = 5 cm) and hatched at 20 ± 0.5 °C (water temperature, L:D = 12 h:12 h). Each clutch usually occupied one container, but a large clutch might be divided into several containers. After their egg yolks were entirely absorbed, larvae were fed a solution of boiled chicken egg yolk once a day for 2 days. Then, tadpoles were fed spirulina powder (China National Salt Industry Corporation, Table 1) once a day, and water was replaced daily. Tadpoles cultured in the same container were defined as a population. These populations ranged from 400 to 1000 individuals. The developmental stages of tadpoles were identified according to the Gosner stage [39].

**Table 1:** Composition of spirulina powder and whole body, liver and tail of stage 30–31 *R. omeimontis* tadpoles. Data are presented as single values or mean ± SE

	Spirulina Powder	Tadpole	Liver	Tail
Total protein (g/100 g)	67.3	7.3	9.54 ± 0.42	–
Sugar (g/100 g)	2.2 ^a^	1.1 ^a^	4.15 ± 0.56 ^b^	0.34 ± 0.07 ^b^
Lipid (g/100 g)	0.6 ^c^	1.3 ^c^	15.0 ± 1.17 ^d^	–
Total energy (kJ/100 g)	1474	186	–	–

^a^ Total sugar content; ^b^ Glycogen content; ^c^ Total lipid content; ^d^ TG content; −---, not detected

**Fig. 1:**
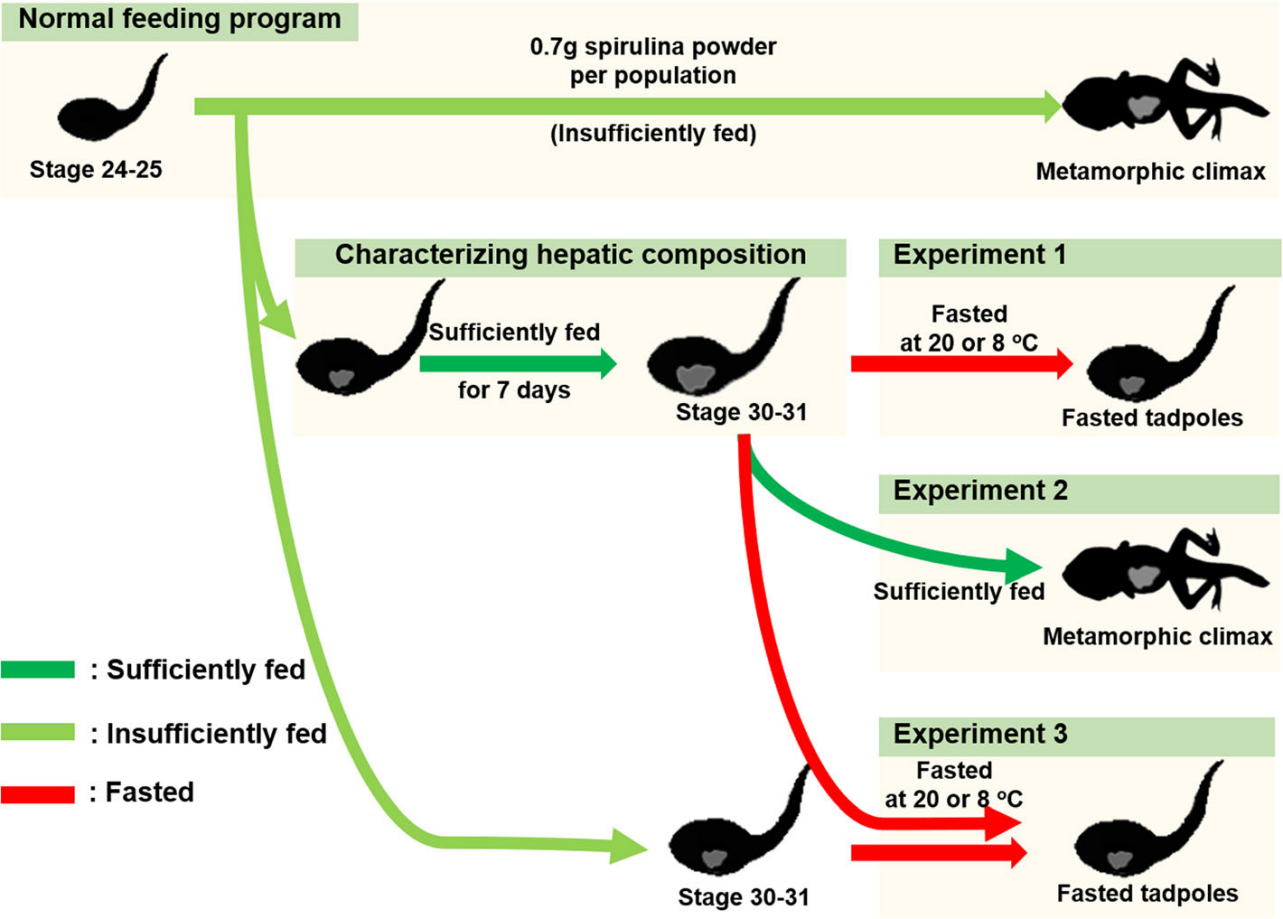
Schematic diagram of the experimental design

*Rana omeimontis* tadpoles are voracious feeders, and their growth rate is positively correlated with the amount of spirulina powder provided daily. Field observation indicated that at least 5–6 months (e.g., from October to May) are required for completing their metamorphosis [38]. If enough food is provided (residual spirulina powder could be observed until next feeding), these tadpoles can complete their aquatic life history within 2 months at 20 ± 0.5 °C (our experience). Tadpole populations kept in our laboratory were normally fed with approximately 0.7 g spirulina powder daily (ground in 10 ml water, an insufficient feeding program) (Fig. 1). Such a feeding program could support the nutrient requirement of tadpole populations with 400–600 individuals to reach their metamorphic climax within 5 months. Increased food availability could improve the liver weight/volume and hepatic fat content of tadpoles. When necessary, tadpoles might be provided with enough food (sufficiently feeding program) to obtain tadpoles with better body condition and more fat deposition. According to our experience, a sufficient feeding program lasting for 7 days was enough to significantly increase the liver weight/volume of insufficiently fed tadpoles and produce typical fatty liver.

### Experimental design

Experiment 1 aimed to reveal the mobilization order of hepatic reserves. Two normally fed tadpole populations (with most individuals reaching stage 29–30) were transferred into a sufficient feeding program. After 7 days, individuals at stage 30–31 were collected (391.5 ± 19.5 mg, mean ± SE). These tadpoles were divided randomly into two groups and fasted at 20 °C (typical temperature in spring) and 8 °C (typical water temperature in winter), respectively. Then, time series sampling (0, 2, 5 and 10 days for 20 °C; 0, 2, 8 and 30 days for 8 °C) was conducted to obtain tadpoles with reducing nutrient stores, and 6–13 individuals were sacrificed from each group at each time point.

Experiment 2 aimed to characterize the role of hepatic fat in metamorphosis. Tadpole populations in Experiment 1 were fed sufficiently until metamorphosis, and individuals were collected at stage 36, 41, 43 and 44 sequentially.

Experiment 3 aimed to reveal the influence of hepatic fat on starvation-induced metamorphosis. Stage 30–31 tadpoles with high hepatic fat accumulation were obtained following the method in experiment 1. Stage 30–31 tadpoles with low hepatic fat accumulation were collected from populations under a normal feeding program. Then, both tadpole groups were starved at 20 and 8 °C, respectively. Then, time series sampling was conducted, and 6–13 individuals were sacrificed for each group at each time point.

### Micro-computed tomography (micro-CT)

After anesthetization by MS-222, tadpoles were fixed in 4% paraformaldehyde for more than 24 h and stained in I_2_ & KI water solutions (respectively, 1% & 2%, w/v) for 12 h. A CT scan was conducted on a Quantum GX Micro CT (PerkinElmer) with the following parameters: scanning current, 70 eV; 10 μM; field of view: 36 × 36 mm for acquisition, 25 × 25 mm for reconstruction; scan duration, 15 min.

### Fat tissue-specific staining

Anesthetized tadpoles were dissected on the abdomen and immersed in 10% (w/v) dithizone-ethanol solution diluted 500-fold with water (with 2% (v/v) ammonia for solubilization) for more than 30 min. Tissues rich in fat were stained dark green, while other tissues were stained orange to red [40].

### Histological sectioning

Anesthetized tadpoles were dissected for collecting liver. These samples were stored in 4% paraformaldehyde until histological analyses. After dehydration in a graded series of ethanol and transparency by xylene, livers were embedded in paraffin and sectioned in serial transverse sections (7 μm thick). Hematoxylin and eosin (H & E) staining, periodic acid-Schiff (PAS) staining and red oil (RO) staining were conducted to show general histological characteristics, neutral lipid content, and glycogen content, respectively.

### Thin-layer chromatography (TLC)

Anesthetized tadpoles were weighed and dissected to collect liver, tail and the rest of the carcass. Weighted tissues were transferred into 2 ml EP tubes with 1 volume Milli-Q water, 2 volumes chloroform and 4 volumes methanol. Then, the tissues were sufficiently homogenized and incubated at 60 °C for 1 h. After centrifugation at 12,000 g for 10 min, 150 μL supernatant was transferred into a clean tube with 50 μL chloroform and 50 μL Milli-Q water, followed by 15 s of vortexing. The mixture was centrifuged at 10,000 g for 10 min to collect the chloroform phase. This solution was dried in a vacuum dryer and dissolved in 30 μL chloroform:methanol (2:1, v/v). Now, the total lipid sample was ready to be analyzed on a silica gel chromatography plate (GF254 100 × 200 mm, AR). After developing with hexane:diethyl ether:acetic acid (60:20:1, v/v), the plate loaded with lipid sample was dried by an air blower and fully sprayed with 10% phosphomolybdic acid in ethanol. Coloration was conducted in an oven at 105 °C for 5 min, and the image of the plate was captured with the Gel Logic 200 Imaging System (Kodak).

### Determination of hepatic glycogen and triglycerides

Anesthetized tadpoles were weighed and dissected to collect the liver. After adding 500 μL 0.1 M PBS (pH 7.4), tissues were homogenized by a tissue grinder, followed by centrifugation at 1000 g for 15 min (4 °C). The supernatant was ready for triglyceride determination. The remaining supernatant (300 μL) was mixed with 600 μL 10% trichloroacetic acid (TCA, w/v). After centrifugation at 1000 g for 15 min, 300 μL of supernatant was transferred into a new tube with 1200 μL of 95% ethanol and allowed to rest overnight. After centrifugation at 1000 g for 15 min, the precipitate was dissolved in 50 μL of water to form the glycogen solution.

The GPO-PAP method was used for triglyceride determination. The method followed the manufacturer’s instructions for the triglyceride assay kit (Nanjing Jiancheng Bioengineering Institute, China). The reaction system included 2.5 μL of supernatant and 250 μL of reagent mix (Tris-HCl, 100 mM; lipase ≥3000 U/L; ATP, 0.5 mM; glycerol kinase, ≥ 1000 U/L; glycerol 3-phosphate oxidase, ≥ 5000 U/L; peroxidase, ≥ 1000 U/L; 4-aminoprotepyrine, 1.4 mM; p-chlorophenol, 3 mM). After incubation at 37 °C for 10 min, the optical density at 510 nm of the mixtures was determined. Glycerol solution (2.26 mM) was used to generate a standard curve.

The reaction system of glycogen measurement included 5 μL of glycogen solution, 85 μL of water and 400 μL of 0.2% anthrone in 80% sulfuric acid. After incubation at 95 °C for 15 min and cooling at room temperature, the optical density at 626 nm of the mixtures was determined. Soluble starch (2 mg/ml) was used to generate a standard curve.

### Metabolomic profiling

After grinding in liquid nitrogen, 100 mg tissue powder was transferred into 1.5 ml Eppendorf tubes with 1 ml methanol:acetonitrile:water = 2:2:1 (v/v), followed by ultrasonication for 30 min × 2 and incubation at − 20 °C for 1 h. After centrifugation at 12,000 for 15 min (4 °C), the supernatants were transferred into new tubes and freeze-dried. Samples were dissolved in 100 μL acetonitrile:water (1:1, v/v) for analysis.

Extracted supernatants were analyzed by LC (1290 Infinity LC, Agilent) coupled with quadrupole-time-of-flight mass spectrometry (Triple TOF 5600+, AB SCIEX). The column was equilibrated with 95% (v/v) solvent A consisting of 25 mM ammonium acetate and 25 mM ammonium hydroxide in Milli-Q water, and 2 μL of each sample was separated by a C18 HILIC column (ACQUITY UPLC HSS T3 1.8 μm, 2.1 mm × 100 mm, Waters) at 25 °C. Separation was performed with 40–95% solvent B (acetonitrile) at 0.3 ml/min as follows: 0–0.5 min, 95% B; 0.5–7 min, decreasing B from 95 to 65%; 7–8 min, decreasing B from 65 to 40%; 8–9 min, 40% B; 9–9.1 min, increasing B from 40 to 95%; 9.1–12 min, 95% B.

Metabolite data were obtained in both positive and negative ion modes with the following settings: ion source gas 1, 60; ion source gas 2, 60; curtain gas: 30; source temperature: 600 °C; ion spray voltage floating, ± 5500 V; TOF MS scan m/z range: 60–1200 Da; product ion scan m/z range: 25–1200 Da; TOF MS scan accumulation time, 0.15 s/spectrum; product ion scan accumulation time, 0.03 s/spectrum. The MS/MS spectra were acquired by information-dependent acquisition with high sensitivity as follows: declustering potential, ± 60 V; collision energy, 30 eV.

Metabolite data were processed using XCMS software (http://metlin.scripps.edu/download/) and Microsoft Excel (Microsoft, Redmond, WA, USA). Data on impurity peaks from column bleeds were excluded. Metabolites were identified by a combination of molecular weight comparison (molecular ion peak) and MS/MS spectrum comparison to a standard library. The relative abundances/concentrations of metabolites are presented as the ion intensities of their molecular ion peaks (Additional file 1).

### Statistical analysis

Hepatosomatic index (HSI) was calculated as the ratio of liver weight to body weight. As the body weight of tadpoles may be variable due to digesta excretion, the hepato-tail index (HTI; the ratio of liver weight to tail weight) was also used to evaluate the relative size of the liver.

Basic statistical analyses were conducted using IBM SPSS v21.0 (IBM, Armonk, NY, USA). Changes in metabolite levels between control and 10-d-fasted tadpoles were analyzed using the independent-sample T test. Since most analyses revealed variation trends in whole groups of metabolites, rather than variations in individual metabolites, no FDR corrections were performed. Differences in body indexes and hepatic reserves between tadpoles with different starvation durations were analyzed using one-way ANOVA, followed by the Student–Newman–Keuls post hoc test. Principal component analysis (PCA) of metabolomes was conducted using Simca-P + 11 (Umetrics AB, Umea, Sweden), with the scaling-type parameter set as ‘Par’. Graphs were created using GraphPad Prism 5 or ggplot2, an R package [41, 42]. Numeric data used in graphs are presented in Additional files 1 and 2.

## Results

### Liver is the primary fat depot in *R. omeimontis* tadpoles

**Fig. 2:**
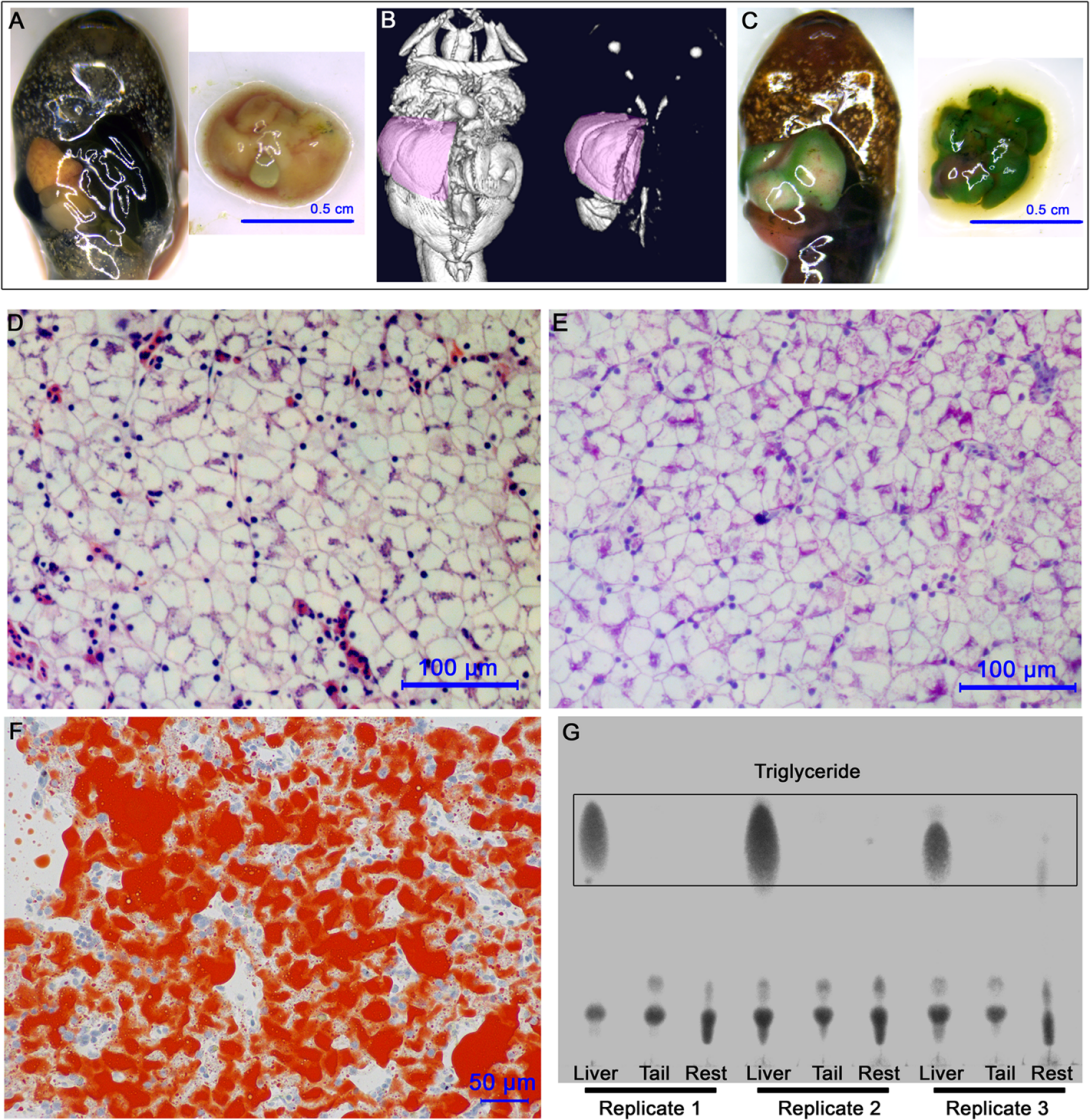
Hepatic fat deposition phenomenon in *R. omeimontis* tadpoles. **a** Typical morphology of a stage 31 *R. omeimontis* tadpole and its liver. **b** Micro-CT imaging of a stage 31 *R. omeimontis* tadpole. **c** Fat-specific stain by dithizone. Tissues with abundant neutral lipids are stained green. The liver is the only organ stained green. **d**-**f** Histological morphology of liver of *R. omeimontis* tadpoles: D, H&E staining; E, PAS staining; F, RO staining. **g** Distribution of triglycerides in *R. omeimontis* tadpoles (by TLC)

*R. omeimontis* tadpoles have no obvious fat body in their larval stages. Instead, a large liver (typically presenting an oily yellow color, Fig. 2a-b) can be detected in the abdominal cavity of tadpoles after Gosner stage 25. For example, the livers of tadpoles at stage 30–31 can account for 7.24 ± 0.23% of the total weight (including the massive intestinal contents) if food is sufficiently provided. After staining with dithizone, these large livers presented a dark green color (Fig. 2c), suggesting the presence of abundant neutral lipids. The histological patterns of their livers resembled the WAT, with enlarged hepatocytes filled by neutral lipid, which resulted in marginalized cell nuclei and glycogen (Fig. 2d-f). According to the results of dithizone staining (Fig. 2c) and TLC analysis (Fig. 2g), the liver is the primary fat depot of *R. omeimontis* tadpoles.

### Metabolite profile of *R. omeimontis* tadpoles

**Fig. 3:**
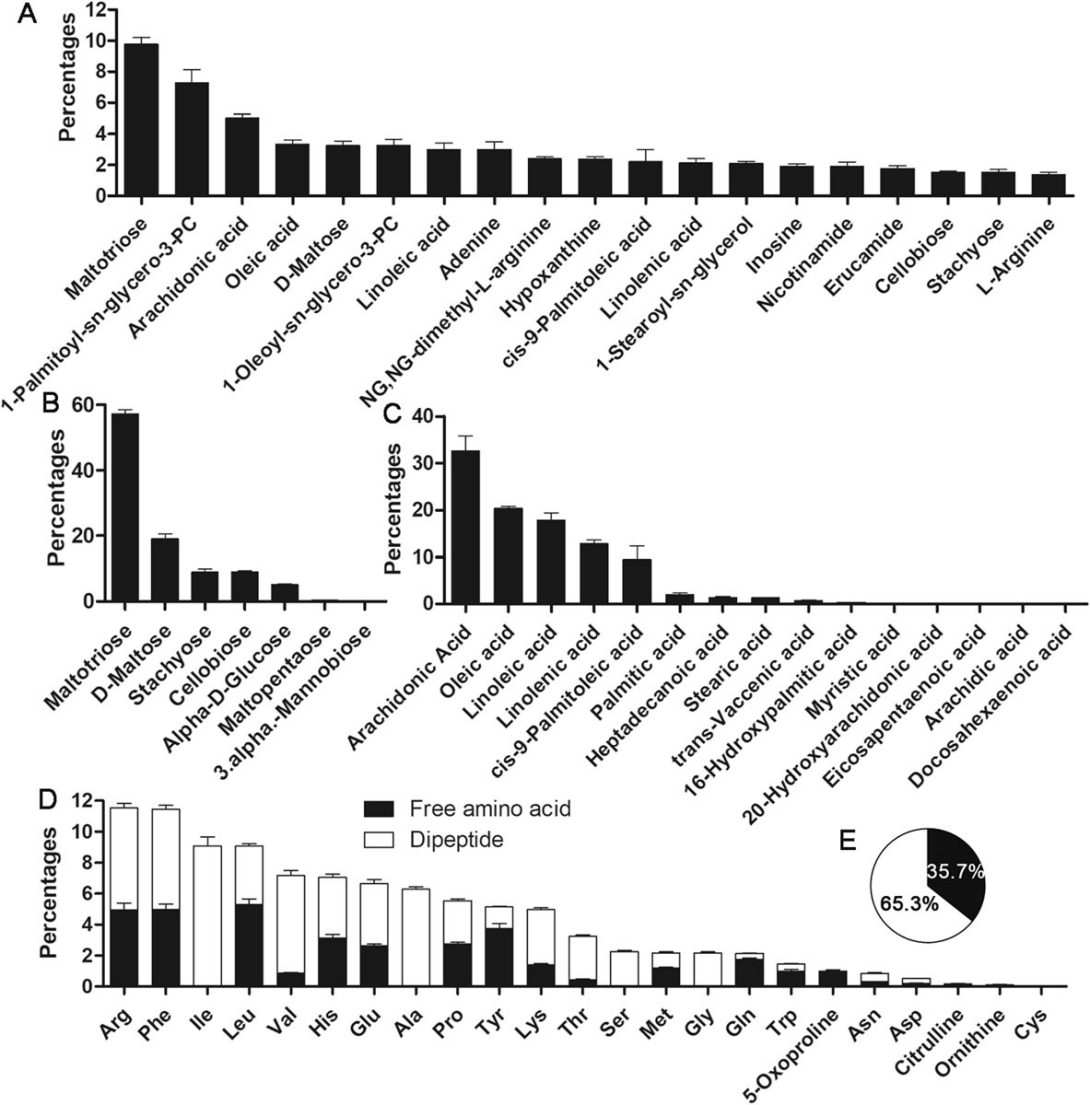
Metabolic profile of liver of *R. omeimontis* tadpoles (Stage 30–31). **a** Metabolites with an abundance higher than 1% of all identified metabolites. **b** Profile of identified soluble carbohydrates. **c** Profile of identified FFAs. **d** Profile of identified amino acids. **e** Proportion of free amino acids and dipeptides in the total amino acid pool. Each column in these two figures represents the mean ± SE of 7 samples

A total of 450 metabolites were identified in *R. omeimontis* liver, and the most abundant metabolites (accounting for > 1% of the total abundance of identified metabolites) were mainly oligosaccharides, FFAs, phospholipids, nucleosides, and amino acids (Fig. 3a). Maltotriose was the dominant carbohydrate in the liver, accounting for 60% of the total carbohydrate (Fig. 3b). Maltose, stachyose and cellobiose also each had a higher abundance than glucose. Fifteen FFAs were identified, and unsaturated ones, including arachidonic acid (20:4Δ^5,8,11,14^), oleic acid (18:1Δ^9^), linoleic acid (18:2Δ^9,12^), γ-linolenic acid (18:3Δ^6,9,12^) and palmitoleic acid (16:1Δ^9^), accounted for more than 93% of the total FFAs in the liver (Fig. 3c). Amino acids were stored as both free (*n* = 18) and dipeptides (*n* = 135) in the liver (Additional file 1), accounting for 35.7 and 64.3% of their sum, respectively (Fig. 3d).

A total of 281 metabolites were identified in the tails. The metabolome of the tail was characterized by a high abundance of carnosine, inosine and creatine (Additional file 3: Figure S1 A), which are related to muscle function. In tails, maltotriose was still the dominant carbohydrate, with much higher abundance than that of glucose (Additional file 3: Figure S1 B). Unlike the liver, the tail had comparable total amounts of saturated and unsaturated FFAs. Stearic acid and palmitic acid were the two most dominant FFAs (Additional file 3: Figure S1 C). Amino acids in tails were mainly in the free form (84.4%) (Additional file 3: Figure S1 D).

### Metabolism of hepatic reserves during starvation

**Fig. 4:**
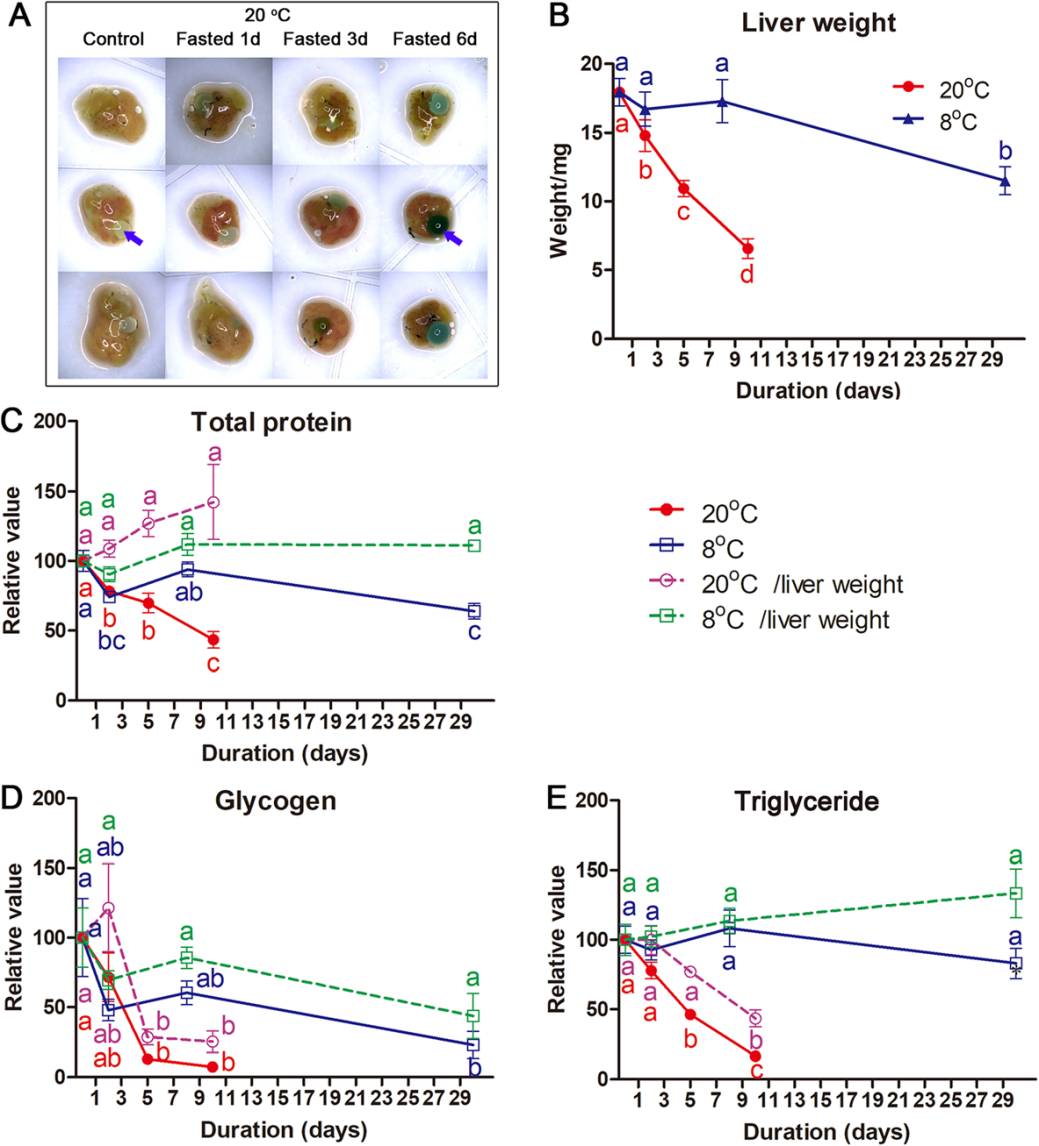
Mobilization of hepatic reserves in fasted *R. omeimontis* tadpoles. **a** Morphological variation of the liver of fasted tadpoles; arrows indicated gall bladders. Change of liver weight (**b**), total protein (**c**), glycogen (**d**) and triglycerides (**e**) during starvation. Each value represents the mean ± standard error (*n* = 6–13). Different letters indicate significant differences between values (*p* < 0.05), as shown by the Student–Newman–Keuls post hoc test after one-way ANOVA

**Fig. 5:**
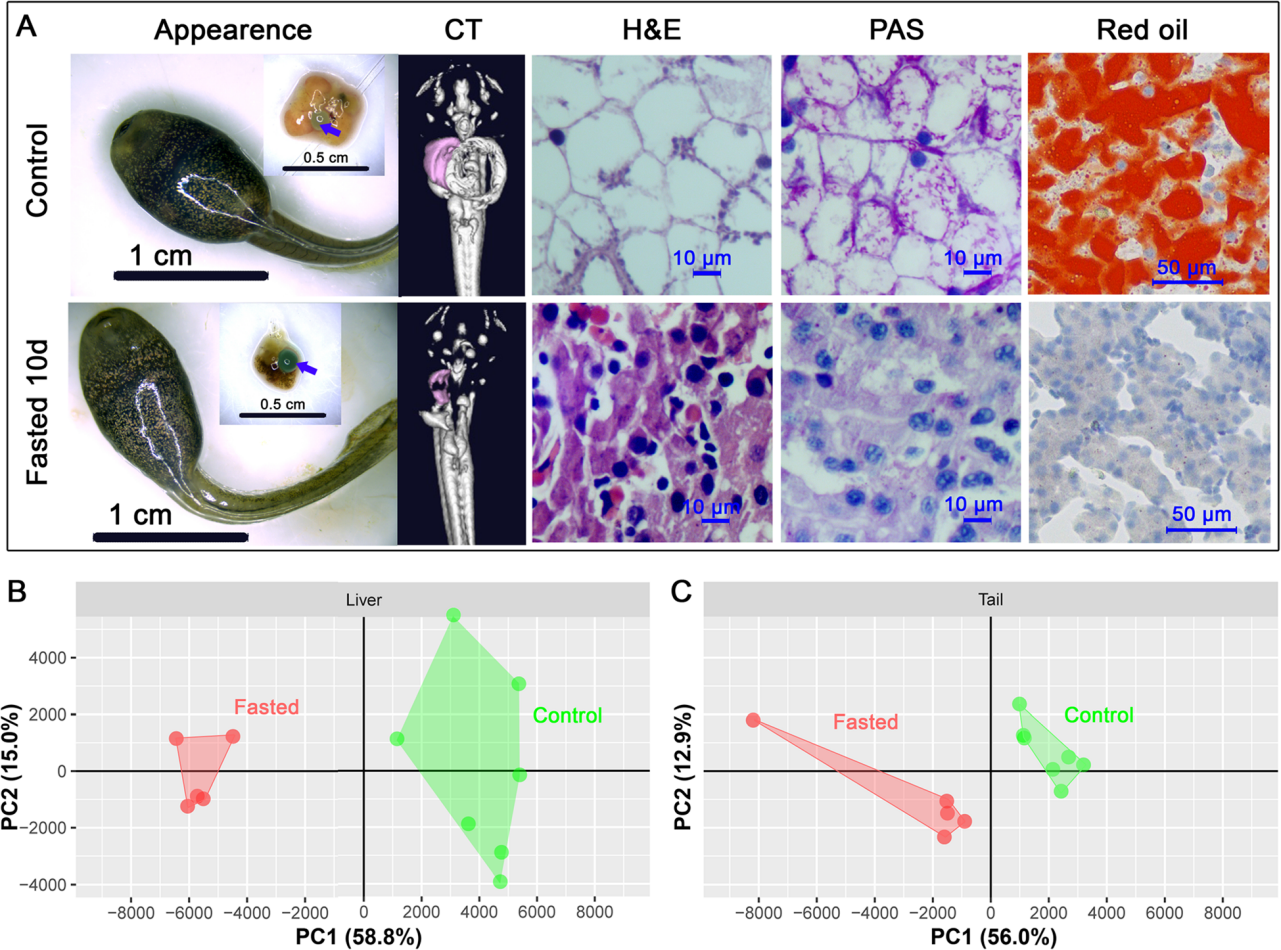
Comparison between fed and 10-d-fasted *R. omeimontis* tadpoles. **a** Comparison of the histological morphology of the liver; arrows indicated two typical gall bladders. **b**-**c** Scatter plots comparing the metabolic profiles of liver (**b**) and tail (**c**) as found by PCA analysis

The liver of *R. omeimontis* tadpoles dwindled in size and weight with the prolongation of fasting, accompanied by color changes from yellow to red and then to brown (Fig. 4a-b). At 20 °C, livers showed rapid weight reduction after fasting, with significant decreases in hepatic protein, glycogen and triglyceride levels (Fig. 4c-e). Glycogen and triglycerides also decreased in content relative to liver weight, suggesting that they were disposable energy reserves. Although glycogen and triglycerides were consumed simultaneously, hepatic glycogen was exhausted within 5 days, while hepatic triglyceride reserves could sustain catabolism more than 10 days. At 8 °C, tadpoles consumed their hepatic resources much more slowly than their 20 °C counterparts (Fig. 4). Glycogen was preferentially consumed as fuel over triglycerides, whose level was unchanged even after 30 days of fasting. After 10 days of fasting at 20 °C, *R. omeimontis* tadpoles consumed most of their hepatic triglycerides. The morphology of their liver changed dramatically (Fig. 5a), to a smaller size and darker color. The liver shifted from an adipose-like histological pattern to that of common hepatocytes, and the diameter of hepatocytes was much smaller due to the disappearance of huge lipid drops (Fig. 5a).

**Fig. 6:**
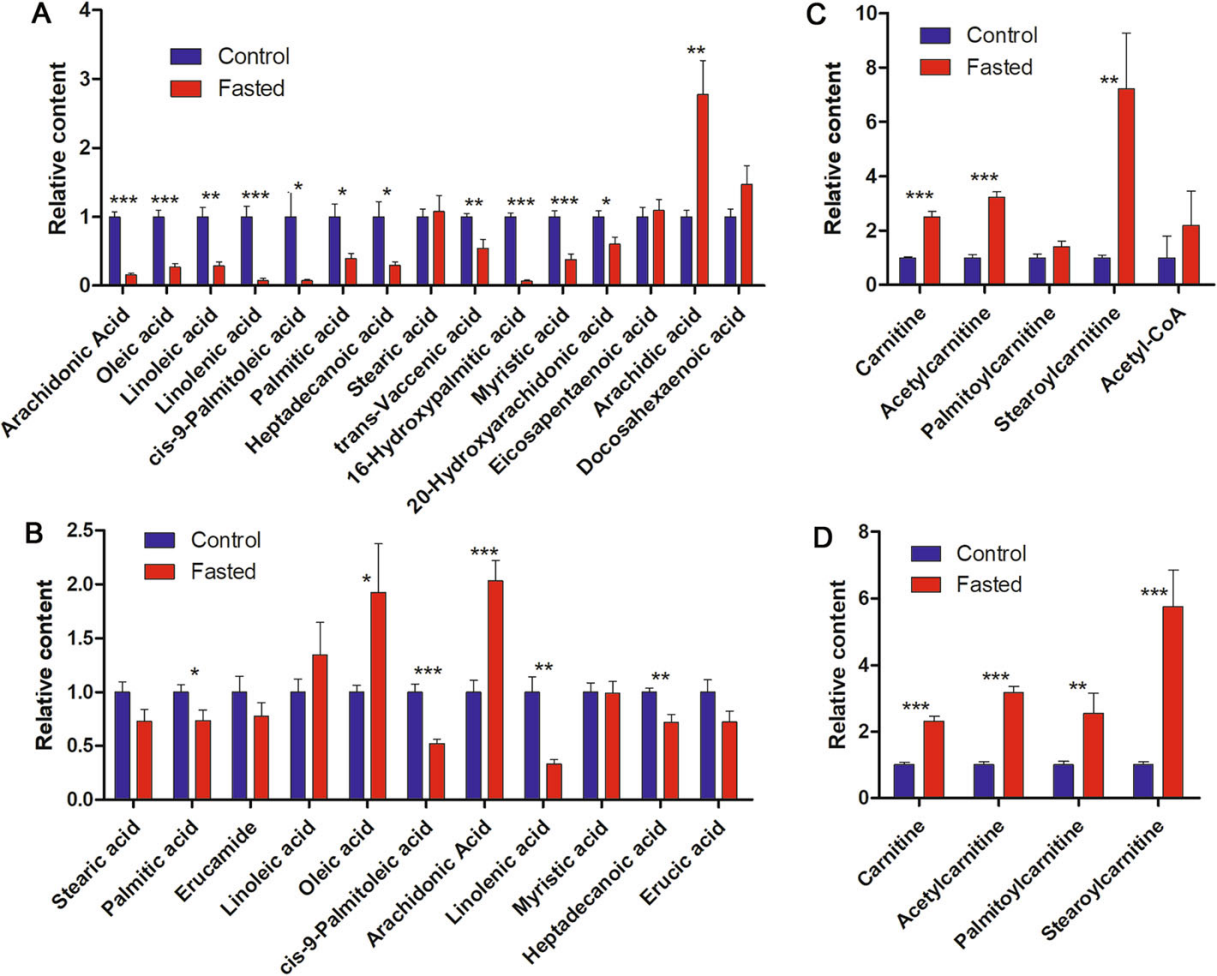
Variation of metabolites involved in lipid metabolism after 10 days of starvation. **a**-**b** Variation of free amino acids in liver **a** and tail **b**. **c**-**d** Variation of intermediates in lipid metabolism in liver **c** and tail **d**. Each column represents mean ± SE (*n* = 5–7), ***: *p* < 0.001, **: *p* < 0.01, *: *p* < 0.05

**Fig. 7:**
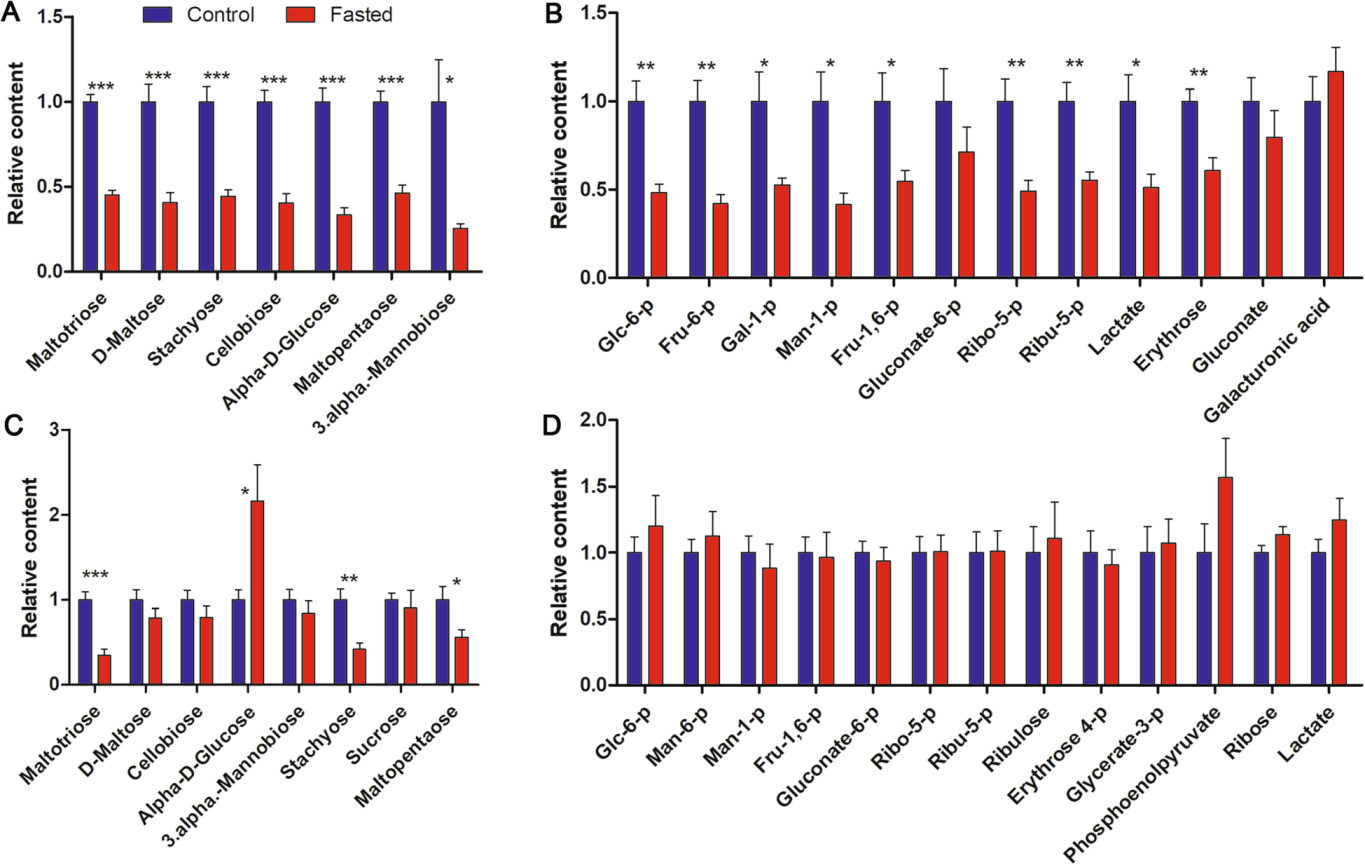
Variation of metabolites involved in carbohydrate metabolism after 10 days of starvation. **a**-**b** Variation of free carbohydrates (**a**) and intermediates in glycolytic pathways (**b**) in liver. **c**-**d** Variation of free carbohydrates (**c**) and intermediates in glycolytic pathways (D) in the tail. Each column represents a mean ± SE (n = 5–7), ***: *p* < 0.001, **: *p* < 0.01, *: *p* < 0.05

**Fig. 8:**
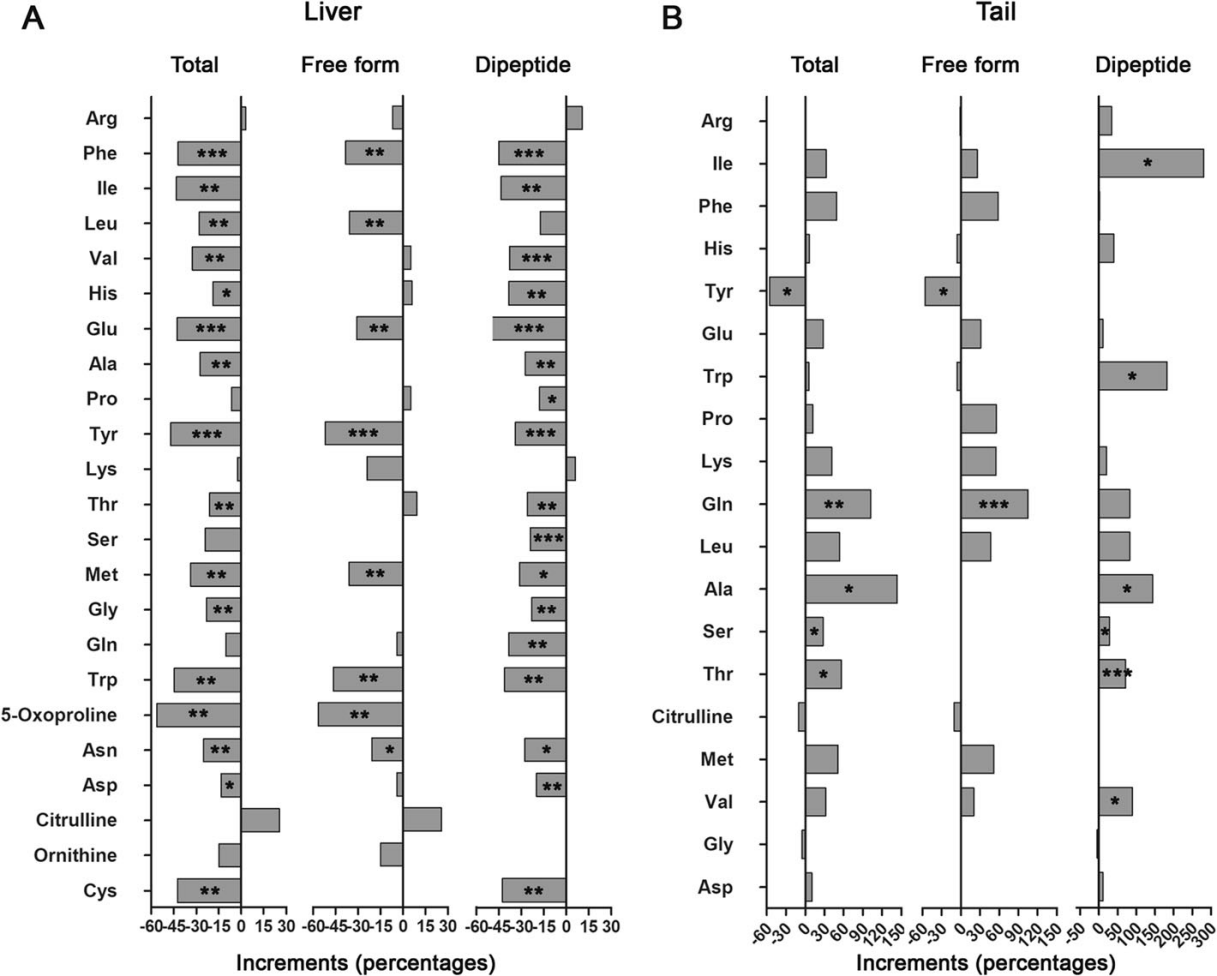
Variation of amino acids after 10 days of starvation. **a** Variation trends of amino acids in liver. **b** Variation trends of amino acids in the tail. Each column represents the percentage change of an amino acid after 10 days of starvation (n = 5–7). ***: *p* < 0.001, **: *p* < 0.01, *: *p* < 0.05

To reveal how hepatic reserves were consumed by tadpoles at 20 °C, liver and tail metabolomes were compared between fed and 10-d-fasted tadpoles. According to the results of PCA, the first principal component (PC1, accounting for 58.8% of the total variance) well divided fed and fasted tadpoles into two groups (Fig. 5b-c). *R. omeimontis* tadpoles consumed most of their major hepatic FFAs after 10 days of starvation (Fig. 6a), with 85, 74, 72, 93, and 93% reductions in arachidonic acid, oleic acid, linoleic acid, linolenic acid and palmitoleic acid, respectively. In the tail, despite palmitic acid, palmitoleic acid, linolenic acid and heptadecanoic acid decreasing, oleic acid and arachidonic acid, the 2 most abundant FFAs in the liver, increased after starvation (Fig. 6b). Increased levels of carnitine, acetyl-carnitine and stearoyl-carnitine were observed after starvation in both liver and tail (Fig. 6c-d), suggesting robust lipid consumption during starvation of tadpoles. Although hepatic glycogen was exhausted after 5 days of starvation, tadpoles fasted for 10 days maintained their hepatic soluble carbohydrates and glycolic intermediates (mainly sugar phosphates) at concentrations 25–50% of those in fed tadpole livers (Fig. 7a-b). In tails, the concentrations of oligosaccharides (maltotriose, stachyose and maltopentaose), disaccharides (maltose, cellobiose, mannobiose and sucrose) and monose (glucose) were decreased, unchanged and increased, respectively (Fig. 7c). The glycolic intermediates in the tail were maintained at comparable levels to those in fed tadpoles even after 10 days of starvation (Fig. 7d). Eight of 18 free amino acids and 70/135 dipeptides decreased significantly (not shown) after 10 days of starvation, resulting in an overall downtrend of total amino acids (16 of 23 decreased, Fig. 8a). In the tail, however, starvation caused an overall uptrend of amino acids in both free and dipeptide forms (Fig. 8b), and the concentrations of glutamine, alanine, serine and threonine showed significant increases (*p* < 0.05). In the aerobic stage, the levels of malate and succinate, two metabolites in the TCA cycle, were decreased significantly in the liver but not in the tail (*p* < 0.05) after 10 days of starvation (Additional file 3: Figure S2), implying a decreased and unchanged consumption rate of metabolic fuels in liver and tail, respectively.

### Variation of hepatic fat in metamorphosis

**Fig. 9:**
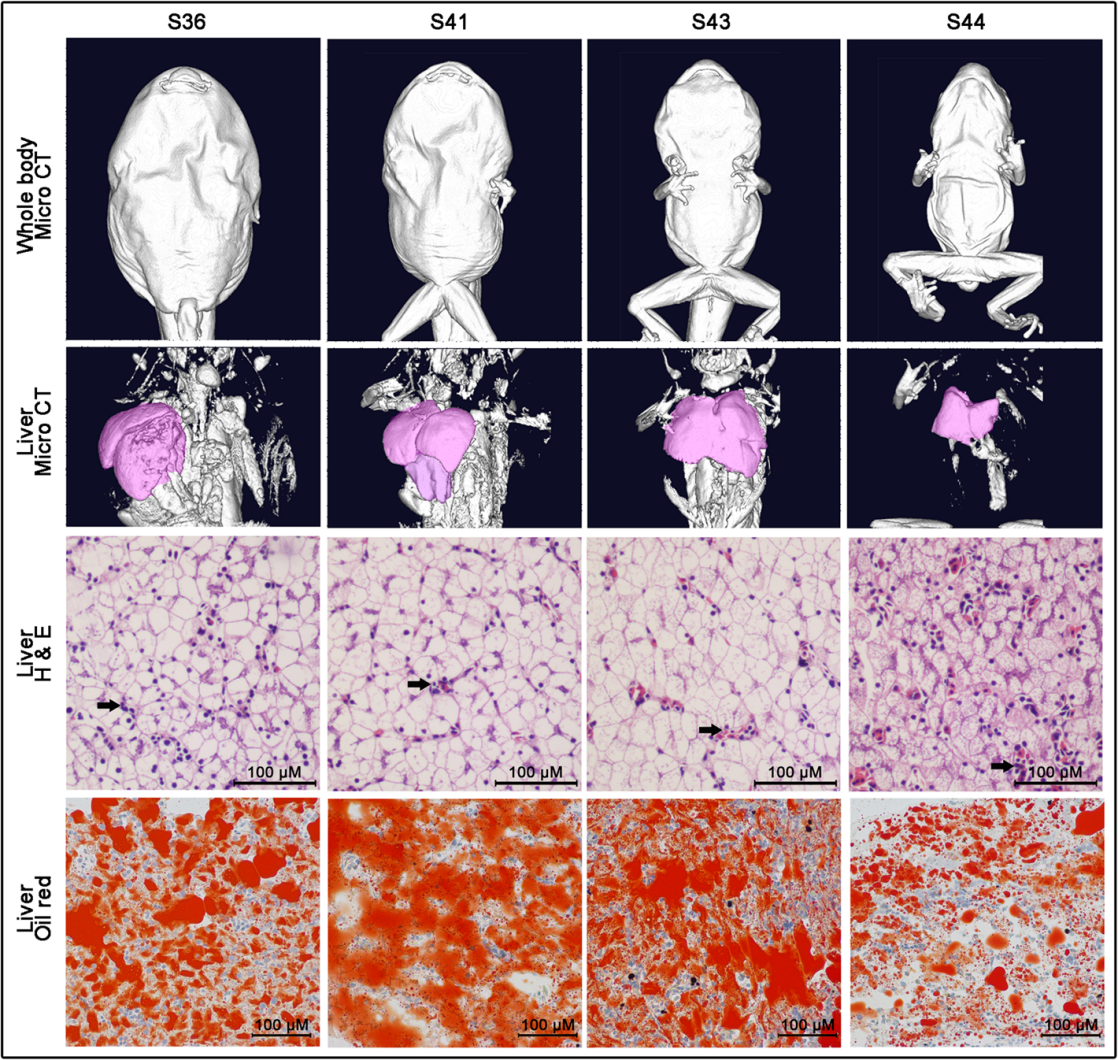
Morphological and histological changes of liver during metamorphosis. Black arrows indicate blood sinus

The liver size, hepatocyte diameter and hepatic fat content did not decrease immediately after the onset of metamorphic climax (after stage 41), and the histological morphology was unchanged from stage 36 to stage 43 (Fig. 9). When the tail of the froglet was absorbed to a stub (stage 44), rapid mobilization of hepatic fat was observed, characterized by a reduction of liver size and hepatocyte diameter, clearance of hepatic fat and higher degree of hepatic vascularization (Fig. 9).

**Fig. 10:**
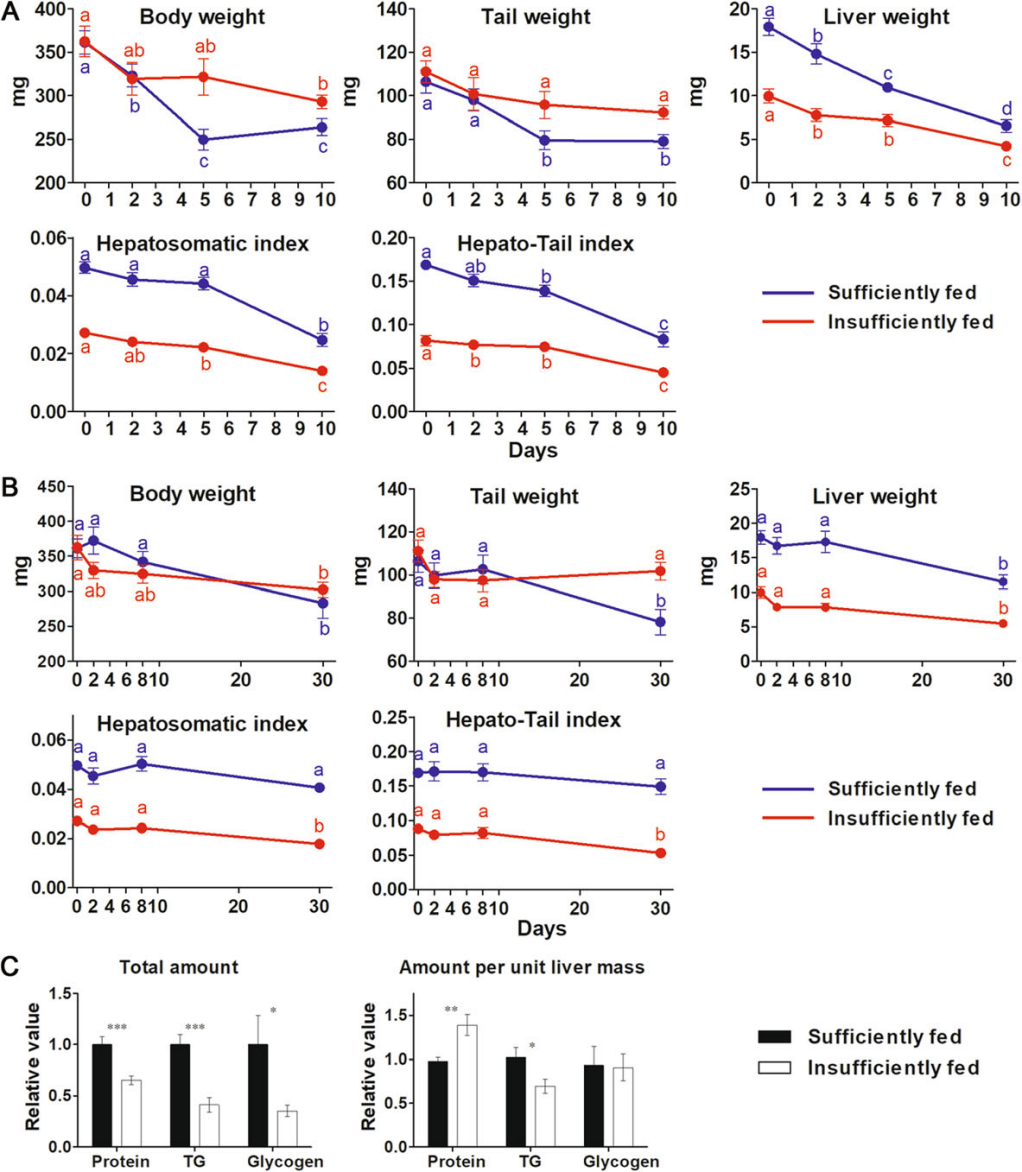
Different responses of sufficiently and insufficiently fed tadpoles to starvation. **a** Variation of body indexes during starvation at 20 °C. **b** Variation of body indexes during starvation at 8 °C. Each value represents the mean ± standard error (n = 6-13). Different letters indicate significant differences between values (*p*  < 0.05), as shown by the Student–Newman–Keuls post hoc test after one-way ANOVA. **c** Comparison of the absolute and relative content of hepatic reserves between sufficiently and insufficiently fed tadpoles. Each column represents mean ± SE (n = 5-7), ***: *p* < 0.001, **: *p* < 0.01, *: *p* < 0.05

Starvation accelerated the appearance of metamorphic traits in sufficiently fed tadpoles at stage 30–31, but not in insufficiently fed ones, even though they had comparable initial body and tail weights (Fig. 10a-j). These metamorphic traits included a dramatic reduction of body size and tail weight and an evacuated and shortened intestine (Fig. 10a-j & Additional file 3: Figure S3). Some individuals also showed tail apoptosis and accelerated development of the hind leg (Additional file 3: Figure S3). Sufficiently fed tadpoles had larger initial liver weight and higher HSI and HTI than the insufficiently fed ones. The contents of hepatic protein, triglycerides and glycogen in sufficiently fed tadpoles were 1.53-fold (*p* < 0.001), 2.43-fold (*p* < 0.001) and 2.83-fold (*p* = 0.028), respectively, higher than those in sufficiently fed tadpoles, while the relative contents of hepatic protein, triglycerides and glycogen in sufficiently fed tadpoles were 0.72-fold (*p* < 0.01), 1.44-fold (*p* = 0.039) and 1.10-fold (*p* = 0.72) of those in insufficiently fed tadpoles.

## Discussion

Using liver as the primary fat depot has not been previously reported for tetrapods. One of the most well-known cases of hepatic fat deposition in tetrapods is ‘foie gras’, the fatty liver of domestic Palmipedes, i.e., ducks and geese. In geese overfed with a carbohydrate-rich diet, the liver weight may increase more than 10-fold, or up to 10% of body weight [43], and their hepatic fat can be comparable to their abdominal fat in content [13]. Even so, the liver of geese should not be as significant as their adipose tissues in fat storage because their artificial fatty liver resulted from compulsive lipogenesis due to excessive carbohydrate intake and was impelled by the limited capacity of fat exportation from liver to peripheral WAT [13]. Normally fed geese store most of their fat in WAT rather than liver [13]. Similarly, although hepatic fat storage is commonly observed in some adult amphibians and reptiles, their liver probably functions more in lipid metabolism (i.e., synthesis, degradation) than in storage [9]. Like hagfish, sharks and cods, *R. omeimontis* tadpoles (and some other frog species) were devoid of fat-accumulated adipose tissues across their larval stage (Fig. 2a & c & g), and thus their liver should be the indispensable fat depot. The hepatic fat deposition phenomenon observed in *R. omeimontis* tadpoles was not an artifact, as was evidenced in individuals caught in the field. Accordingly, to the best of our knowledge, this was the first observation that liver can be the primary fat depot in tetrapods.

### Potential protective mechanisms against hepatic injury in *R. omeimontis* tadpoles

*Rana omeimontis* tadpoles store body fat mainly in their liver (Fig. 1). The histological morphology of their fatty liver is characterized by inflated polyhedral hepatocytes filled by lipids. It differs from the histology of pathological fatty liver in two ways. First, hepatic fat in pathological fatty liver presents as round droplets, which usually inflate and sphericize hepatocytes, while hepatic fat in *R. omeimontis* tadpoles is shaped into polygons by the hepatocytes. Second, the lipid droplets in pathological fatty liver vary greatly in size, resulting in obvious morphological asymmetry among hepatocytes. This is consistent with previous studies showing a similar histological morphology of Atlantic cod liver [44]. It seems that naturally fatty livers can better manage their hepatic fat than pathological livers, which could be a potential strategy to avoid liver injuries caused by fat deposition. To some extent, the histology of *R. omeimontis* tadpoles resembles WAT in cell morphology. This makes sense, as fat deposition in the liver is likely a primitive trait in vertebrate evolution [2]. This finding also implied that WAT and primitive fatty liver may share similar molecular strategies for cellular fat management. It is interesting to speculate that the liver has lost the capacity to manage massive cellular lipids during the evolution of vertebrates. In future studies, comparative lipidomics and proteomics should be conducted to investigate the differences among lipid droplets from naturally fatty liver, from pathological fatty liver, and from WAT.

In the progression of nonalcoholic steatosis hepatitis in humans, lipotoxicity can induce initial hepatic injuries, which are then amplified by inflammation [14]. However, hepatic lipid accumulation is not inevitably accompanied by lipotoxicity, whose occurrence depends on the efficiency of incorporating FFA into triglycerides and the composition of intermediates of lipid metabolism in liver [33, 45]. Previous studies have indicated that the ratio of monounsaturated FFA to saturated FFA determines whether liver cells are damaged by the flux of FFA in mammals, and a higher ratio indicates a lower risk of hepatic stress [45, 46]. *R. omeimontis* tadpoles kept low proportions of saturated FFAs (1.98% for palmate and 1.28% for stearate), but they exhibited much higher proportions of monounsaturated ones (19.5% for oleate and 12.99% for palmitate) in their liver FFA pool (Fig. 3c). Such a metabolic trait is expected to be associated with a low risk of lipotoxicity. Abundant free arachidonic acid is another prominent feature of the hepatic FFA pool of *R. omeimontis* tadpoles (Fig. 3c). This FFA has been suggested as a protective metabolite against lipotoxicity induced by saturated FFAs through its diversion of saturated FFAs into triglycerides [47–49]. As the precursor of both proinflammatory and anti-inflammatory eicosanoids [50], arachidonic acid may also protect the liver from damage by regulating inflammation. Overall, the FFA composition in *R. omeimontis* tadpole liver is beneficial to avoid lipotoxicity and inflammation. Interestingly, neither a high abundance of arachidonic acid nor a high ratio of monounsaturated FFAs to saturated FFAs was observed in the tail metabolome of these tadpoles (Additional file 3: Figure S1 C), suggesting that these metabolic traits are specific to the liver. We speculated that two approaches might be involved in maintaining this unique hepatic FFA profile. One possible approach is effective FFA desaturation in the liver. So far, many studies have indicated that the activity of stearoyl-CoA desaturase-1, the enzyme that converts saturated FFAs to unsaturated FFAs, is associated with the susceptibility of hepatocytes to lipoapoptosis induced by saturated FFAs [30, 51]. It will be meaningful to determine the expression level of stearoyl-CoA desaturase-1, as well as its upstream regulators, in hepatocytes of *R. omeimontis* tadpoles. Another possible approach is the selective transport of FFAs to peripheral organs [52]. In contrast to the situation in the liver, the proportions of saturated FFAs in the tail are much higher than those of monounsaturated FFAs (Additional file 3: Figure S1 C). Considering that tail FFAs also come from the liver, the huge difference in FFA profile between liver and tail imply selective export of saturated FFAs from the liver to other organs. Such a mechanism, if confirmed in the liver of *R. omeimontis* tadpoles, is meaningful in the treatment of pathological fatty liver.

### Metabolic traits of fatty liver of *R. omeimontis* tadpoles

There are several prominent differences in the metabolic profile between *R. omeimontis* tadpoles and other invertebrates. The first is an unusually high abundance of maltotriose but a low abundance of glucose (the common blood sugar of vertebrates) in the liver and tail of *R. omeimontis* tadpoles (Fig. 3b). We measured the metabolic composition of spirulina and excluded the tadpole diet as the source of maltotriose (Supplementary data). We speculate that maltotriose may also exist in the circulation of *R. omeimontis* tadpoles. Previous studies have indicated low blood sugar in amphibians, which usually have blood sugar levels below 25 mg/dl [53]. The values in other vertebrates, including lamprey, elasmobranchs, bony fish, birds and mammals, range between 45 and 200 mg/dl [54]. If disaccharides or trisaccharides indeed accounted for a considerable proportion of blood sugars in amphibians, the total carbohydrate amount would be comparable to that in other vertebrates. This is because the methods used to measure the concentration of blood sugar in these studies were either specific to glucose or indiscriminate to reducing sugars. Therefore, both methods could underestimate the mass of disaccharides and trisaccharides in blood. It would be interesting to know why *R. omeimontis* tadpoles use maltotriose as their major soluble sugar in liver and whether it is associated with their hepatic fat deposition phenomenon. One plausible explanation is that the hydrophilic space in their hepatocytes is scarce, and maltotriose is more efficient than glucose (at the same concentration) at transporting carbons. The second metabolic difference between *R. omeimontis* tadpoles and other vertebrates is the existence of numerous and abundant dipeptides in liver (Fig. 3d). Although some dipeptides, such as carnosine and anserine, are common in vertebrate tissues such as muscle and brain [55, 56], these metabolites have not been reported as the major storage form of soluble amino acids in vertebrates. In liver of *R. omeimontis* tadpoles, some amino acids, including isoleucine, alanine, serine and glycine, are only stored in the form of dipeptides. This metabolic trait is seemingly specific to liver, as the tail has a much lower proportion of dipeptides and fewer dipeptide types. The physiological significance of dipeptides in liver of *R. omeimontis* tadpoles is still unknown. We may again speculate that their prevalence is related to the scarce hydrophilic space in hepatocytes.

Though elasmobranchs, Atlantic cod and *R. omeimontis* tadpoles share the same fat storage strategy, the utilization orders of their fat reserves are quite different. In elasmobranchs, their hepatic fat is neither a prior metabolic fuel nor a prior substrate for ketogenesis to amino acids [57]. In Atlantic cod, fat is only a metabolic fuel at the beginning of a fasting period, a phenomenon that is also observed in bony fish with extrahepatic fat depots [7, 37, 44, 58]. In *R. omeimontis* tadpoles, their liver glycogen and fat are mobilized simultaneously at 20 °C, but glycogen is exhausted more rapidly than fat (Fig. 4). After 10 days of starvation, the decrements in hepatic protein and amino acids were less severe than that of triglycerides or FFAs (Figs. 4, 6 and 8). These results suggested a sequential utilization of hepatic reserves in *R. omeimontis* tadpoles: glycogen > lipids > protein/amino acids. This order is in accordance with those in fasted frogs [59–62] and mammals [63, 64]. Their carbohydrates are mainly used at the beginning of a long-term fasting period, and then they depend on their lipids and protein for energy production. For *R. omeimontis* tadpoles, temperature could influence the utilization of hepatic reserves. At 8 °C, glycogen was preferentially consumed over fat. Such a metabolic change might result from cold-induced suppression of aerobic metabolism, which has been reported in overwintering amphibians. This is because they can suppress their aerobic metabolism to cope with cold temperatures [29, 65]. As oxidation of FFAs is an obligative aerobic process, fat mobilization is likely more effectively inhibited. The huge gap in consumption rates (more than 3-fold for all three hepatic reserves, Fig. 4) between tadpoles fasted at 20 and 8 °C also implied the existence of metabolic suppression. It should be noted that a decrease in glycogen (to less than 4% of liver weight) could not explain the reduction of liver weight (more than 30% of liver weight) in *R. omeimontis* tadpoles fasted at 8 °C (Fig. 4). According to our observation, the reduction in blood volume, induced by starvation and evidenced by the accumulation of biliverdin (a product of heme catabolism, Fig. 4a), should make a great contribution.

How reserves are allocated among tissues is also an important aspect of metabolism. In animals with extrahepatic depots, FFAs released from these depots are mainly used by liver and muscle during starvation. This was also the case in *R. omeimontis* tadpoles, as the increment of carnitine and acyl-carnitine concentrations suggested enhanced lipid catabolism in both liver and muscle after starvation (Fig. 6c-d). Unlike these metabolic intermediates, fatty acids were part of lipid storage in liver of *R. omeimontis* tadpoles, which can be expected to reduce in liver (Fig. 6a). Despite the dramatic decrement in hepatic FFAs detected after 10 days of starvation, the levels of major tail FFAs were maintained (Fig. 6b). This was because the tail was a major consuming organ, which could import fatty acids from the blood circulation. In the tail, the concentrations of arachidonic acid and oleic acid, the two FFAs with the highest abundance in the liver, increased significantly after 10 days of starvation. Palmitic acid, cis-9-palmitoleic acid, linolenic acid and heptadecanoic acid, which also had relatively high abundance in the liver, decreased significantly after 10 days of starvation. Such a variation pattern supported the encouraged transport of hepatic FFAs to the tail and the increased utilization of FFAs in the tail. In fasted mammals, glycogenolysis and gluconeogenesis based on glycerol and amino acids were activated in their liver to maintain the levels of blood sugar. The maintenance of a high amount of hepatic soluble sugars, even 5 days after glycogen exhaustion, suggesting that gluconeogenesis was activated in the liver of fasted *R. omeimontis* tadpoles (Fig. 7a). Therefore, carbohydrates were unlikely metabolic fuels in the liver of fasted tadpoles. As a major consuming organ, the tail was likely a destination of the carbohydrate from the liver, as its levels of glycolytic intermediates remained unchanged even 5 days after glycogen exhaustion (Fig. 7d). In addition, the variation pattern of tail soluble carbohydrates, which could be explained by the encouraged hydrolysis of oligosaccharides to monoses (Fig. 7c), could also suggest the consumption of carbohydrates in the tail. Considering that liver is the primary site for gluconeogenesis and that amino acids are major substrates for gluconeogenesis, gluconeogenesis in liver likely accounted for the decrement of hepatic amino acids in fasted *R. omeimontis* tadpoles (Fig. 8a). In contrast to the liver, the tail had an overall increment in amino acid concentrations. We thought these variations likely resulted from protein degradation, a common metabolic response to long-term starvation in vertebrates [35]. It was interesting that the concentrations of alanine and glutamine, two major nontoxic interorgan ammonia carriers [66, 67], had the most prominent increments (Fig. 8b), implying catabolism of amino acids in the tail. Taken together, these data suggest that lipids and possibly amino acids were used as metabolic fuel in the liver of fasted *R. omeimontis* tadpoles, while lipids, carbohydrates and amino acids were all used as metabolic fuel in their tail. In combination with the variation trends of metabolites in the TCA cycle, these results suggest that the metabolic flux throughout aerobic pathways was likely decreased and maintained in the liver and tail, respectively, after 10 days of starvation. It seemed that the tail had the priority of maintaining energy production to the liver during starvation. This is reasonable, as a large proportion of energy produced in liver is used for anabolism, which is likely suppressed in starvation, while locomotion is always required for animals in foraging and escaping.

Overall, *R. omeimontis* tadpoles have some unique features in their liver metabolome, and these features likely correspond to their liver-based fat deposition. *R. omeimontis* tadpoles share similar metabolic strategies with amphibians and mammals that have extrahepatic fat depots. This implies that the fatty liver of *R. omeimontis* tadpoles may be a primitive counterpart of mammal WAT.

### Role of hepatic fat in metamorphic climax

Although hepatic fat of *R. omeimontis* tadpoles can be mobilized immediately during starvation (Fig. 4e), the onset of metamorphic climax, a nonfeeding period, was not accompanied by mobilization of hepatic fat (Fig. 9). This finding suggested that metabolic reserves other than hepatic fat supported energy production in metamorphosing *R. omeimontis* tadpoles. After the tail was absorbed to a stub, a dramatic decrease in hepatic fat was observed in these tadpoles, suggesting that apoptotic tissues were used as metabolic substrates in early metamorphic climax. Our results also indicated the existence of a metabolic shift in metamorphic climax, which is consistent with observations in the tadpole of the bullfrog (*Lithobates catesbeianus*), whose fat body lipid is also mobilized in late metamorphic climax [23]. Our results suggest that the liver or hepatic fat is functionally equal to the fat body or fat body lipid, respectively, in fueling metamorphosis. Importantly, the metabolic shift in metamorphic climax could reconcile the controversy over whether fat reserves are required for energy fuel in metamorphosing tadpoles. Although lipid reserves have been suggested to be reduced after metamorphosis [20], several studies have indicated that tadpoles with low lipid reserves can also complete metamorphosis [26, 68]. According to our results based on *R. omeimontis* tadpoles, fat reserves might not be necessary for the major stage of metamorphic climax, as organ remodeling was mainly completed when the tail of *R. omeimontis* tadpoles was absorbed to a stub. However, fat reserves should be important in supporting the energy metabolism in these froglets [23], as their physiological functions might be incompetent to ensure a predation success rate. Additionally, mobilization of fat reserve in late metamorphic climax might relieve the dependency on amino acids for catabolism and thus facilitate more amino acids flowing into anabolism. These speculations were supported by observations in salamanders, in which low lipid levels during metamorphosis were associated with a low survival rate of metamorphosed animals [25].

Environmental stress can accelerate the metamorphosis of tadpoles by inducing the release of corticosteroids [69–71]. Food deprivation is a common environmental stress in the wild, and its influence on the progression of tadpole metamorphosis is associated with developmental stage and external conditions [23, 72, 73]. It has been widely reported that food deprivation likely accelerates development in pro-metamorphic tadpoles in a condition-dependent manner [74–78]. Crespi and Denver [76] speculated that fat stores in tadpoles should have a role in modulating the strength of food deprivation-induced corticosterone signals and that tadpoles with abundant fat stores likely respond weakly to food deprivation. Our results also suggest a role for energy reserves in modulating accelerated metamorphosis induced by food deprivation. However, nutrient storage was positively correlated with the inducibility of accelerated metamorphosis in fasted *R. omeimontis* tadpoles (Fig. 10). We speculated that energy reserves could be a body condition signal that is independent of the corticosterone cascade in modulating metamorphosis. This is supported by observations in *Xenopus laevis* tadpoles, in which increased leptin expression in the fat body just before the onset of metamorphosis serves as a body condition signal to coordinate the feeding activity and the onset of metamorphic climax [24]. In our opinion, stress signals and body condition signals are both indispensable in mediating accelerated metamorphosis induced by food deprivation. This speculation may provide an explanation for the absence of accelerated metamorphosis in pro-metamorphic tadpoles suffering food deprivation [72, 73]. In our study, despite all three energy reserves (triglyceride, glycogen and protein) being higher in the livers of sufficiently fed tadpoles than in those of insufficiently fed ones, triglycerides were most likely responsible for this suppositional body condition signal in regulating metamorphosis for several reasons. First, triglycerides are the major hepatic energy reserve in *R. omeimontis* tadpoles, and they have a role in fueling metamorphic climax. Second, the liver triglyceride and glycogen contents in sufficiently fed tadpoles were both twice the values in insufficiently fed tadpoles, but the content of liver glycogen in sufficiently fed tadpoles varied greatly, exceeding a 90-fold difference between individuals. Lastly, liver triglycerides were the only reserve that had higher relative content in sufficiently fed tadpoles than insufficiently fed ones. Further studies are required to verify and characterize this suppositional body condition signal in *R. omeimontis* tadpoles, and it will be interesting to test whether leptin can still be a signal messenger of fat stores in the liver.

## Conclusion

In this study, we reported a hepatic fat deposition phenomenon in *R. omeimontis* tadpoles. To our knowledge, this was the first observation that liver can be the primary fat depot in animals with a higher evolutionary status than bony fish. We found that this naturally fatty liver had special histological morphology and metabolic compositions, which likely either guard the liver against injuries or make hepatocytes of *R. omeimontis* tadpoles adapt to fat accumulation. During starvation, *R. omeimontis* tadpoles shared similar metabolic traits with frogs and mammals that have extrahepatic fat depots. This finding suggested that their fatty liver could be a primitive counterpart of mammal WAT. In addition, we found that the fatty liver of *R. omeimontis* tadpoles had roles in fueling and even modulating metamorphosis.

## Supplementary information

**Additional file 1.** Liver and tail metabolite tables.

**Additional file 2.** Numeric data used in graphs.

**Additional file 3: Figure S1.** Metabolic profile of the tail of *R. omeimontis* tadpoles (Stage 30–31). (A) Metabolites with an abundance higher than 1% of all identified metabolites. (B) Profile of identified soluble carbohydrates. (C) Profile of identified FFAs. (D) Profile of identified amino acids. (E) Proportion of free amino acids and dipeptides in the total amino acid pool. Each column in these two figures represents the mean ± SE of 7 samples. **Figure S2.** Variation of intermediates in the TCA cycle after 10 days of starvation in liver (A) and tail (B). Each column represents mean ± SE (*n* = 5–7), ***: *p* < 0.001, **: *p* < 0.01, *: *p* < 0.05. **Figure S3.** Sufficiently fed tadpoles showed traits of accelerated metamorphosis after starvation. Major morphological traits of accelerated metamorphosis included evacuated and shortened intestine, tail apoptosis (rounded tail) and accelerated development of hind limb (toe development). These three traits did not appear in any tadpoles before starvation treatment (either sufficiently fed or insufficiently fed ones), as well as in insufficiently fed tadpoles after starvation.

## Data Availability

The dataset supporting the conclusions of this article is included as additional file.

## Abbreviations

FFA: Free fatty acid
H & E: Hematoxylin and eosin
HSI: Hepatosomatic index
HTI: Hepato-tail index
PAS: Periodic acid-schiff
PC: Principal component
PCA: Principal component analysis
RO: Red oil
TG: Triglyceride
TLC: Thin-layer chromatography
WAT: White adipose tissue
