# Supplementary material for: Characterizing the composition, metabolism and physiological functions of the fatty liver in Rana omeimontis tadpoles
Source: Front Zool. 2019 Nov 14;16:42. doi: 10.1186/s12983-019-0341-x (PMC6854647; doi:10.1186/s12983-019-0341-x)
Supplement: Supplementary file 3 — Additional file 3: Figure S1. Metabolic profile of the tail of R. omeimontis tadpoles (Stage 30–31). (A) Metabolites with an abundance higher than 1% of all identified metabolites. (B) Profile of identified soluble carbohydrates. (C) Profile of identified FFAs. (D) Profile of identified amino acids. (E) Proportion of free amino acids and dipeptides in the total amino acid pool. Each column in these two figures represents the mean ± SE of 7 samples. Figure S2. Variation of intermediates in the TCA cycle after 10 days of starvation in liver (A) and tail (B). Each column represents mean ± SE (n = 5–7), ***: p < 0.001, **: p < 0.01, *: p < 0.05. Figure S3. Sufficiently fed tadpoles showed traits of accelerated metamorphosis after starvation. Major morphological traits of accelerated metamorphosis included evacuated and shortened intestine, tail apoptosis (rounded tail) and accelerated development of hind limb (toe development). These three traits did not appear in any tadpoles before starvation treatment (either sufficiently fed or insufficiently fed ones), as well as in insufficiently fed tadpoles after starvation. [file 12983_2019_341_MOESM3_ESM.pdf]

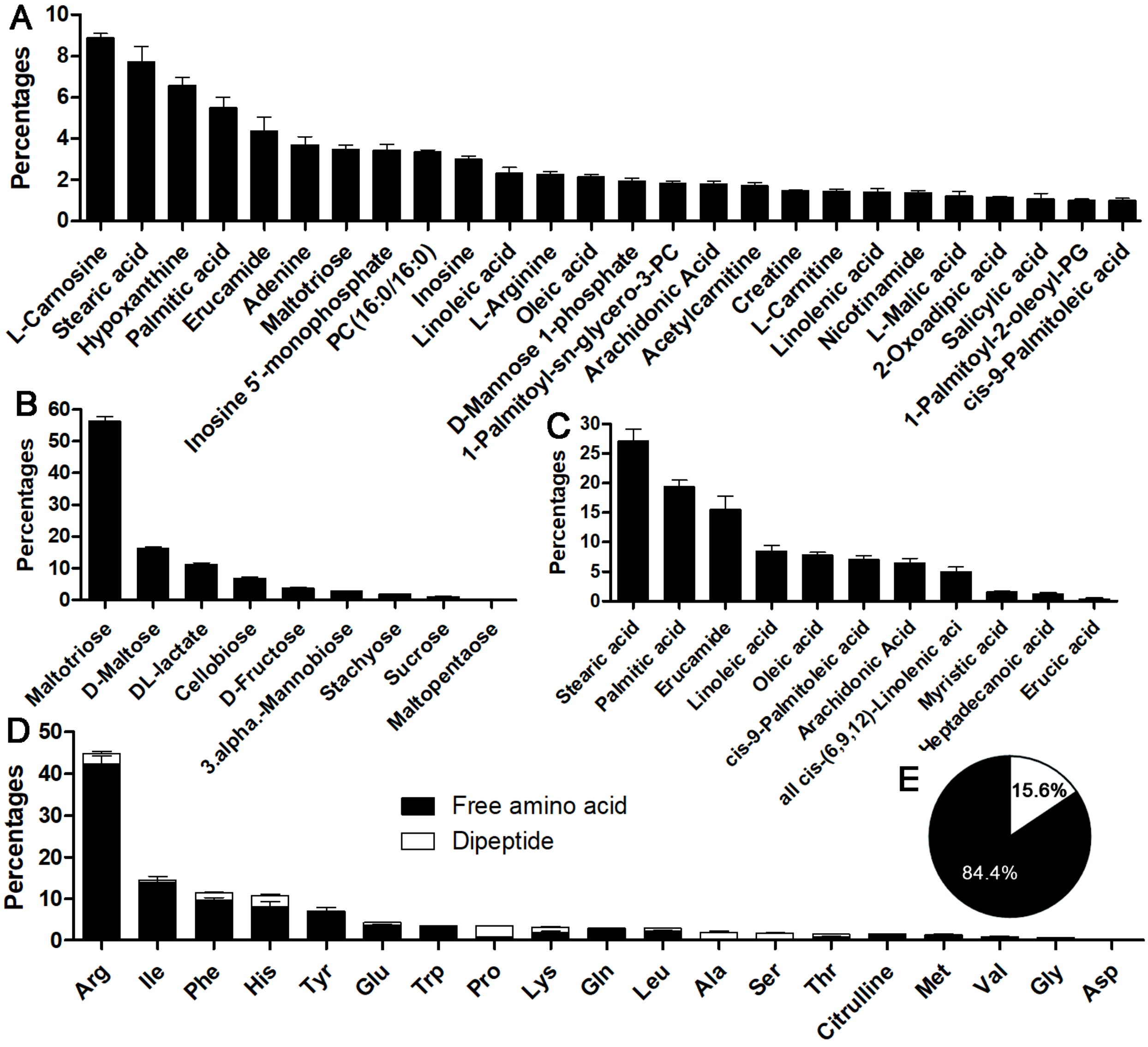

Figure S1 Metabolic profile of tail of *R. omeimontis* tadpoles (Stage 30-31). (A) Metabolites with abundance higher than 1% of total abundance of all identified metabolites. (B) Profile of identified soluble carbohydrates. (C) Profile of identified FFAs. (D) Profile of identified amino acids. (E) Proportion of free amino acids and dipeptides in total amino acid pool. Each column in these two figures represents the mean ± SE of 7 samples.

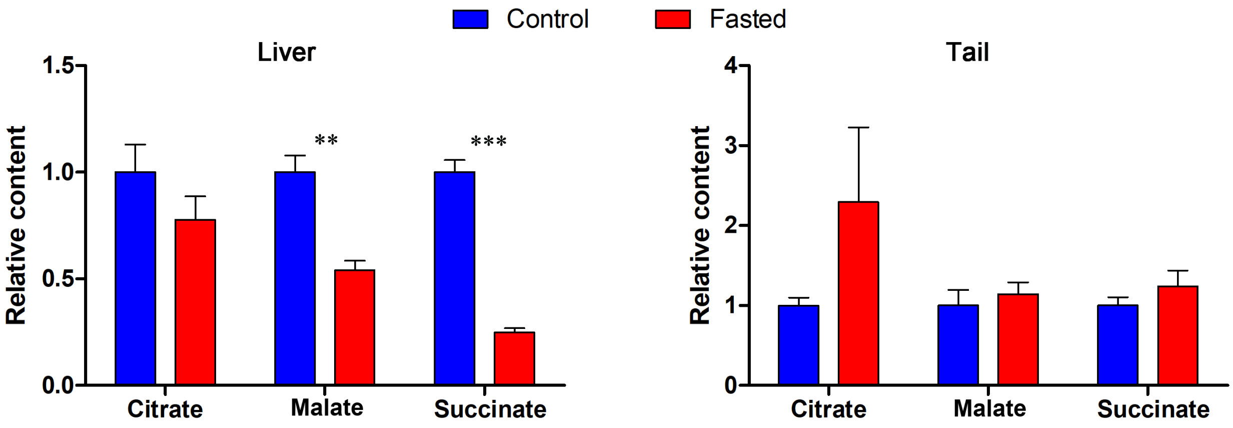

Figure S2 Variation of intermediates in TCA cycle after 10 days of starvation in liver (A) and tail (B). Each column represents a mean ± SE (n = 5-7), ***: $p < 0.001$, **: $p < 0.01$, *: $p < 0.05$.

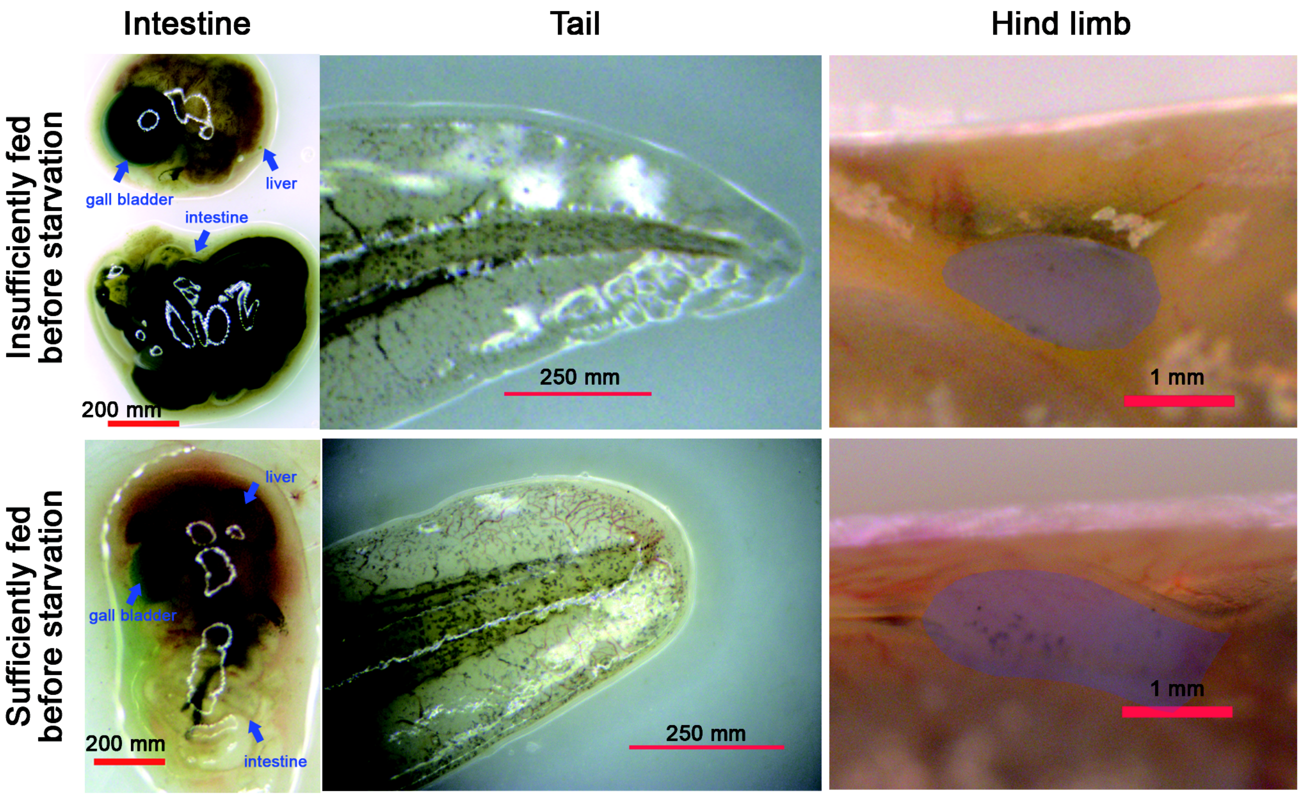

Figure S3 Sufficiently fed tadpoles showed traits of accelerated metamorphosis after starvation. Major morphological traits of accelerated metamorphosis included evacuated and shortened intestine, tail apoptosis (rounded tail) and accelerated development of hind limb (toe development). These three traits did not appear in any tadpoles before starvation treatment (either sufficiently fed or insufficiently fed ones), as well as in insufficiently fed tadpoles after starvation.
